# Biochemical Responses of *Anopheles* spp. Larvae to a Novel Brazilian BR101 *Bacillus thuringiensis* var. *israelensis* Formulation: Oxidative Stress, Detoxification Enzymes, and Safety for Nontarget Notonectidae and Gerridae Aquatic Insects

**DOI:** 10.1002/cbdv.202502891

**Published:** 2025-12-08

**Authors:** Izabel Cristina de Oliveira Bentes, Dayane Dantas Abensour, Maria Luiza Lima da Costa, Raquel Telles de Moreira Sampaio, Leticia Bernadete da Silva, Francisco Augusto da Silva Ferreira, Cláudia Patrícia da Silva Tavares, Hergen Vieira de Souza, Francisco de Assis Marque, Mário Antonio Navarro da Silva, Eduarda Andrade de Lima, Gislayne Trindade Vilas‐Boas, João Antonio Cyrino Zequi, André Correa de Oliveira, Rosemary Aparecida Roque

**Affiliations:** ^1^ Laboratório de Controle Biológico e Biotecnologia da Malária e da Dengue Instituto Nacional de Pesquisas da Amazônia Manaus Amazonas Brazil; ^2^ Laboratório de Etnoepidemiologia Instituto Nacional de Pesquisas da Amazônia Manaus Amazonas Brazil; ^3^ Departamento de Biologia Animal e Vegetal Centro de Ciências Biológicas Programa de Pós‐Graduação Em Ciências Biológicas da Universidade Estadual de Londrina Londrina Paraná Brazil; ^4^ Departamento de Química Universidade Federal do Paraná Curitiba Paraná Brazil; ^5^ Departamento de Zoologia Universidade Federal do Paraná Curitiba Paraná Brazil; ^6^ Departamento de Biologia Geral Centro de Ciências Biológicas Universidade Estadual de Londrina Londrina Paraná Brazil

**Keywords:** *Bacillus*, Culicidae, eco‐friendly, malaria, oxidative

## Abstract

Despite its proven efficacy, *Bacillus thuringiensis israelensis* (Bti) has not yet been incorporated into Brazilian Ministry of Health programs targeting *Anopheles* spp., the primary malaria vectors. This study evaluated the larvicidal potential of the Brazilian strain BR101. The strain displayed significant activity, with mortality rates ranging from 11% ± 2% to 91% ± 5% (LC_50_ = 3.13 µg/mL), accompanied by increased reactive oxygen species (54.67 ± 3 µmol H_2_O_2_), lipid peroxidation (57.33 ± 4.5 ηmol), and oxidative protein damage (16.67 ± 2.1 nM reactive carbonyls/mg). Biochemical responses included elevated activities of superoxide dismutase (29.00 ± 3 mU/mg protein), catalase (17.00 ± 1 µmol H_2_O_2_), glutathione peroxidase (29.00 ± 3 mmol NADPH/min/mL), mixed‐function oxidases (11.00 ± 3 nmol cytochrome/mg protein), and esterases (*α*: 20.67 ± 2; *β*: 25.67 ± 1 µmol/min/mg). Acetylcholinesterase activity was slightly reduced (80.33 ± 7 µmol/min/mg). Ecotoxicological assays revealed no lethal effects on nontarget aquatic predators (Notonectidae, Gerridae), with 100% survival over 30 days, equivalent to controls. These findings demonstrate that Bti BR101 is effective against *Anopheles* larvae while being safe for nontarget organisms.

## Graphical abstract

Bti BR101 exhibits strong larvicidal activity against *Anopheles* larvae spp., with dose‐dependent mortality and LC_50_ of 3.13 µg/mL. Bti increases ROS, lipid and protein oxidation, and activates antioxidant enzymes (SOD, CAT, GPx) and detoxification pathways (MFO and esterases), indicating moderate oxidative stress. Importantly, Bti shows no lethal effects on non‐target aquatic predators, supporting its safety and suitability for vector control.

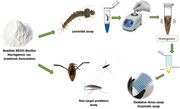

## 1 Introduction

Malaria remains one of the most significant parasitic diseases affecting public health in Latin America, particularly in Brazil, where it is endemic to the Amazon region, which accounts for approximately 99% of all reported cases [1]. According to recent data, by June 2025, a total of 21 706 cases had been recorded in the Brazilian Amazon [2].

Of these cases, 84.78% were caused by *Plasmodium vivax* Grassi & Feletti, 1890 and 15.22% by *P. falciparum* Welch, 1897 (Plasmodiidae), both transmitted primarily by mosquitoes of the genus *Anopheles*, particularly *An. darlingi* Root, 1926, the main vector in the region [2, 3]. However, secondary species, such as *An. nuneztovari* Gabaldón, 1940, *An. triannulatus* Neiva & Pinto, 1922, *An. albitarsis* Lynch Arribálzaga, 1878, *An. aquasalis* Curry, 1932, and *An. braziliensis* Chagas, 1907, can also act as vectors [4, 5, 6, 7].

In Brazil, malaria transmission is strongly influenced by ecological determinants such as high temperatures, elevated humidity, and the presence of aquatic habitats suitable for mosquito oviposition [8]. These conditions favor the proliferation of *Anopheles* spp., leading to increased vector density and, consequently, higher transmission risk [9]. Additionally, socioeconomic factors, including limited access to healthcare, inadequate sanitation, and activities such as illegal mining and logging in forested areas, intensify human exposure to infected mosquitoes [7, 8, 9, 10, 11, 12].

As part of mosquito vector control efforts, Brazil primarily relies on chemical strategies targeting both immature and adult mosquito stages at breeding sites, using insecticide formulations from the pyrethroid and neonicotinoid classes [13, 14]. Although pyrethroids are effective against certain populations of mosquitoes, their intensive and repeated use has led to the development of resistance in others and poses significant ecotoxicological risks to nontarget aquatic organisms [15, 16, 17, 18, 19].

Given the environmental risks and resistance issues associated with synthetic insecticides, the Brazilian Ministry of Health recommends the use of biological larvicides based on *Bacillus thuringiensis* subsp. *israelensis* (Bti) strains (Goldberg, L.J. & Margalit, J.) [20]. Currently, however, Bti formulations are applied exclusively to control *Aedes aegypti* Linnaeus, 1762 (Culicidae) [21]. This limited scope highlights an underexplored opportunity, as extending Bti‐based interventions to *Anopheles* species could provide a safe and effective alternative for malaria vector control in Brazil [22].

Although Brazil has not yet implemented Bti strains for *Anopheles* spp. control, several studies have demonstrated their larvicidal efficacy against major malaria vectors. For example, Panneerselvam et al. [23] revealed that Bt achieved LC_50_ values between 1.72 and 2.42 g/L against larvae of *An. stephensi* Liston, 1901 under both laboratory and semi‐field conditions. Similarly, Demissew et al. [24] reported 100% mortality of resistant *An. arabiensis* Patton, 1905 when treated with Bti Barjac, 1978 (strain AM65‐52) at doses as low as 0.05 g/m^2^ within 24 h. A third study, Ebadollahi et al. [25], exploring other *B. thuringiensis* subsp. *jegathesan* Krieg, Huger & Langenbruch, 1983 isolates, also confirmed significant larval mortality in *An. stephensi* across diverse instar stages.

Beyond larval mortality, Bt strains may also induce physiological stress, particularly through mechanisms involving oxidative imbalance [24]. Although Bt does not directly activate the same oxidative pathways triggered by synthetic insecticides, its Cry and Cyt toxins disrupt gut epithelial integrity and may exacerbate internal stress, suggesting potential synergistic effects that merit further biochemical investigation [26].

For example, several studies have shown that exposure to Bt can elicit oxidative stress responses in both target and nontarget insect species. Moderate doses of the Cry1Ac toxin caused significant cellular damage in the midgut of *Drosophila melanogaster* Meigen, 1830 (Drosophilidae) larvae, including shortened microvilli, enlarged vacuoles, and mitochondrial dysfunction, accompanied by elevated levels of reactive oxygen species (ROS), reduced ATP synthesis, and decreased aconitase activity, confirming the role of Cry toxins in mitochondrial disruption and oxidative stress induction [27].

Similarly, *Galleria mellonella* Linnaeus, 1758 larvae infected with *B. thuringiensis* ssp. *galleriae* strain 69‐6 exhibited increased antioxidant enzyme activity, including superoxide dismutase (SOD) and glutathione (GSH) *S*‐transferase, elevated oxidized thiols and malondialdehyde (MDA), alongside reduced catalase (CAT) activity [20]. Additionally, exposure to the mycotoxin zearalenone amplified ROS production and mortality in *Caenorhabditis elegans* under Bt infection, further implicating oxidative stress in the mode of action [28].

Although these findings were not obtained from *Anopheles* spp. neither with Bti strains or toxins with toxicity to Diptera, they suggest that exposure to Bti may also activate similar oxidative stress pathways in mosquito larvae, with Cry and Cyt toxins not only disrupting the gut epithelium but also potentially inducing oxidative damage that contributes to larval lethality [20].

Given the well‐established larvicidal activity of Bti against Diptera species and the lack of its application by the Brazilian Ministry of Health in programs targeting *Anopheles* spp., we developed a novel formulation based on the Brazilian strain BR101. This strain, isolated from tropical environments, has demonstrated superior larvicidal efficacy and persistence against *Culex quinquefasciatus*, maintaining high biological activity even under semi‐field conditions as thoroughly outlined in our research [29, 30].

These characteristics indicate that BR101 combines strong entomopathogenic potential with remarkable environmental adaptability attributes that distinguish it from conventional Bti strains and make it a particularly promising candidate for *Anopheles* control under Amazonian conditions [23].

Importantly, the adoption of BR101‐based formulations could address a critical gap in Brazil's malaria control strategies, as no biological larvicide is currently available for *Anopheles* management. Thus, beyond representing a scientific advance, BR101 offers a strategic and environmentally sustainable alternative for malaria vector control in the Amazon region.

On the basis of these premises, the present study investigates the larvicidal potential of BR101 against *Anopheles* spp., considering their surface‐feeding behavior. In addition, it examines whether larval exposure to this strain induces oxidative stress as a secondary physiological response, providing further insight into its mode of action beyond midgut epithelial disruption.

## 2 Materials and Methods

### 2.1 Chemical and Reagents

All solvents and reagents used in this study were purchased from Sigma‐Aldrich (St. Louis, MO, USA) and Merck (São Paulo, Brazil), including dimethyl sulfoxide (DMSO), α‐cypermethrin, potassium phosphate buffer (0.1 M, pH 7.3), potassium iodide (KI), hydrogen peroxide standard solution (250 mM), trichloroacetic acid (TCA, 10%), thiobarbituric acid (TBA, 0.5% w/v), 2,4‐dinitrophenylhydrazine (DNPH, 10 mM in 2 M HCl), ethanol, ethyl acetate, guanidine hydrochloride (6 M), formic acid (50%), Bio‐Rad protein assay reagent (diluted 1:5), HEPES buffer (20 mM, pH 7.0), sodium phosphate buffer (0.025 M with 0.1 mM EDTA, pH 10.0), tetramethylethylenediamine (TEMED), quercetin (0.15% in dimethylformamide), reduced GSH, NADPH, glutathione reductase (GR), sodium acetate (3 M), 3,3′,5,5′‐tetramethylbenzidine (TMBZ), hydrogen peroxide (3%), α‐naphthyl acetate (0.3 mM), β‐naphthyl acetate (0.3 mM), Fast Blue dye (30 mg), sodium dodecyl sulfate (SDS, 3%), 5,5′‐dithiobis‐(2‐nitrobenzoic acid) (DTNB, 10 mM), and acetylthiocholine iodide (AChI, 8 mM).

### 2.2 Produce and Formulation the Bti Strain BR101

For the present study, Bti BR101, lot no. T85E2, with a toxicity of 2.700 ITU/mg and presenting 2.2 × 10^8^ CFU, was used. The Bti strain BR101 used in the bioinsecticide preparation was sourced from the Entomopathogenic Bacteria Bank of the Laboratory of Bacterial Genetics and Taxonomy, Department of General Biology, Biological Sciences Center, State University of Londrina, located in Londrina, Paraná, Brazil. The methodology for culturing and formulating as dispersible granules using this Brazilian strain is comprehensively described in our previous publication [29, 30].

### 2.3 Collection of *Anopheles* spp. Larvae

*Anopheles* spp. larvae were collected using entomological dippers, following the guidelines outlined in the Malaria Entomology Manual for Entomology and Vector Control Technicians published by the Pan American Health Organization [31]. Collections were conducted in a fish farming pond located at Ramal Brasileirinho (3°0′42.9″ S, 59°52′29.9″ W), Manaus, Amazonas, Brazil.

Sampling focused on semi‐shaded areas rich in marginal vegetation, particularly macrophytes, which provide *Anopheles* spp. larvae with protection against predators. The collected larvae were then transferred using Pasteur pipettes into 10 mL Falcon tubes containing 5 mL of water from the breeding sites. Once collection was complete, the larvae were transferred into 10‐L containers containing 5 L of water and vegetation from their original breeding sites.

These containers were then transported to the Laboratory of Biological Control and Biotechnology of Malaria and Dengue, where the larvae were acclimatized for 24 h under controlled conditions (temperature: 26°C ± 3°C, relative humidity: 85%, and a 12:12 h light/dark photoperiod) and subsequently identified using a taxonomic key [32].

### 2.4 Larvicidal Assay

Larvicidal assay was carried out following the Guidelines for Laboratory and Field Testing of Mosquito Larvicides from World Health Organization [31]. Groups of 10 *Anopheles* spp. (*n* = 250) larvae, previously identified using taxonomic key [32], were distributed in five recipients (1 L) containing 498 mL of distilled water and concentrations from 0.7 to 9 µg/mL previously prepared in 2 mL of DMSO. As positive and negative controls, these were used: α‐cypermethrin (0.010–0.060 µg/mL) and DMSO (0.7–9 µg/mL). The assay was performed in quintuplicate.

Larval mortality was assessed at 72 h post‐treatment. The percentage of larval mortality was calculated using the formula: larvicidal activity (%) = (number of dead larvae/number of larvae used) × 100. The relative potency (RP) was identified using the following equation: RP = LC_50_ of standard larvicidal/LC_50_ of Bti strain BR101 [33].

### 2.5 Biochemical Assays

#### 2.5.1 Preparation of Larval Homogenate

At the conclusion of the larvicidal bioassay, *n* = 100 *Anopheles* spp. larvae from each treatment group, Bti strain BR101, DMSO at 9 mg/mL, and α‐cypermethrin at 0.0006 mg/mL, were individually collected and distributed into eight sterile 5 mL tubes. Each sample was homogenized in 2.5 mL of ice‐cold potassium phosphate buffer (0.1 M, pH 7.3) using vortex agitation for 5 min to ensure thorough cellular disruption. The homogenates were then subjected to centrifugation at 4000 rpm for 5 min at 4 °C to separate the soluble protein fraction.

The resulting supernatants, containing enzyme‐rich, were carefully transferred to sterile 3 mL microcentrifuge tubes and stored at 4°C until enzymatic activity assays were performed. Biochemical analyses were conducted in triplicate, and absorbance measurements were recorded using a microplate reader set to specific wavelengths corresponding to the optimal detection range for each enzyme evaluated. For each treatment group, biochemical analyses were conducted using three independent biological replicates, each analyzed in technical triplicate to ensure reproducibility and accuracy of the measurements [16].

#### 2.5.2 Measurement of ROS

To estimate ROS levels, particularly hydrogen peroxide (H_2_O_2_), a colorimetric assay was employed based on the method originally by Velikova et al. [34] with modifications adapted for larval supernatants [35]. In brief, 45 µL of each larval supernatant sample was combined with an equal volume (45 µL) of 10 M potassium iodide (KI). The reaction mixture was incubated, and absorbance was subsequently recorded at 390 nm using a microplate spectrophotometer. H_2_O_2_ concentrations were determined by interpolating absorbance values against a calibration curve constructed from serial dilutions of a 250 mM H_2_O_2_ stock solution, ranging from 0 to 45 µmol. Final values were reported as µmol of H_2_O_2_ per gram. All measurements were carried out in technical triplicates to ensure reproducibility.

#### 2.5.3 Lipid Peroxidation Damage

Lipid peroxidation was evaluated by determining MDA levels, a principal marker of oxidative damage to lipids, using the thiobarbituric acid reactive substances (TBARS) method, originally established by Buege and Aust [36], and subsequently adapted for larval homogenates [16]. For the assay, 125 µL of each larval homogenate was combined with 250 µL of a solution containing 10% TCA and 0.5% (w/v) TBA. The resulting mixtures were incubated, and absorbance was measured at 535 nm. To correct for sample turbidity, readings at 600 nm were subtracted. The analyses were conducted using a microplate reader, and MDA concentrations were normalized and expressed as nanomoles per gram of tissue (ηmol/g). All procedures were performed in triplicate, and data were presented as a percentage relative to the control group.

#### 2.5.4 Protein Oxidation Damage

To evaluate oxidative damage to proteins, the method proposed by Levine et al. [37] was utilized, which is based on the detection of carbonyl groups generated by oxidative stress. These groups arise from the interaction of hydroxyl radicals, produced via H_2_O_2_, with protein residues. The carbonyl groups react with DNPH, forming 2,4‐dinitrophenylhydrazone, a compound that can be quantified spectrophotometrically. For the assay, 100 µL of larval supernatant were incubated with 150 µL of DNPH solution (10 mM in 2 M HCl) for 1 h. Subsequently, 250 µL of 6% TCA were added to precipitate the proteins, followed by centrifugation at 1000 rpm for 5 min. The resulting pellet was washed three times with a 1:1 ethanol and ethyl acetate solution to remove excess reagents. After washing, the pellet was resuspended in 1 mL of 6 M guanidine hydrochloride in 50% formic acid. The absorbances were read at 366 nm, and the results were expressed as nM of reactive carbonyls/mg protein. All procedures were performed in triplicate.

#### 2.5.5 Measurement of Total Protein

Total protein concentrations were measured by adding 300 µL of Bio‐Rad reagent (diluted 1:5) to 10 µL of each supernatant, resulting in a final volume of 310 µL per well. Absorbance was then measured at a wavelength of 620 nm as described [38].

### 2.6 Enzymatic Assays

#### 2.6.1 SOD Activity

SOD activity was measured according to the protocol of Kostyuk and Potapovitch [39], with adaptations by Janner et al. [40] for mosquito larval supernatants. For sample preparation, larval supernatants were mixed with 250 µL of 20 mM HEPES buffer (pH 7.0) and centrifuged at 15 000 rpm for 10 min at 4°C to obtain clarified extracts. The assay was carried out in a reaction medium composed of sodium phosphate buffer (0.025 M, 0.1 mM EDTA, pH 10.0) and TEMED. The reaction was initiated by the addition of 0.15% quercetin dissolved in dimethylformamide. The inhibition of quercetin auto‐oxidation was monitored at 406 nm for 2 min using a spectrophotometer. SOD activity was calculated on the basis of the amount of protein required to achieve 50% inhibition of oxidation, and results were expressed in milliunits per milligram of protein (mU/mg protein). All measurements were performed in triplicate.

#### 2.6.2 CAT Activity

CAT activity was assessed by monitoring the degradation of H_2_O_2_ at 240 nm, following the method established by Aebi [41] and later modified by Abolaji et al. [42]. For each larval supernatant, the reaction was carried out in triplicate using a mixture composed of 20 µL of sample, 180 µL of 300 mM H_2_O_2_, and 1.8 mL of 0.1 M phosphate buffer (pH 7.3) at 25°C. The decrease in absorbance was recorded, and CAT activity was expressed as µmol of H_2_O_2_ decomposed per minute per mg of protein.

#### 2.6.3 Glutathione Peroxidase (GPx) Activity

GPx activity was determined on the basis of the method of Paglia and Valentine [43], with modifications by Olufemi‐Salami et al. [44] adapted for larval supernatants. The reaction system consisted of reduced GSH, NADPH, and GR in phosphate buffer. The enzymatic reaction was initiated by the addition of 100 µL of hydrogen peroxide (H_2_O_2_) to the reaction mixture. The consumption of NADPH was monitored at 340 nm over 3 min using a UV–Vis spectrophotometer. All assays were conducted in triplicate, and GPx activity was expressed as millimoles of NADPH oxidized per minute per milliliter (mmol NADPH/min/mL).

#### 2.6.4 Mixed‐Function Oxidase (MFO) Activity

The activity of MFOs was determined using a colorimetric assay adapted from the protocol by Vale et al. [45], originally developed to assess insecticide resistance in *An. darlingi*. To prepare the necessary reagents, a 0.25 M sodium acetate buffer (pH 5.0) was obtained by diluting 41.6 mL of 3 M sodium acetate (NaOAc) into 450 mL of distilled water, with pH adjusted accordingly. The chromogenic substrate solution was prepared by dissolving 10 mg of TMBZ in 5 mL of methanol, followed by the addition of 15 mL of the acetate buffer. Each reaction well in a 96‐well microplate received 200 µL of the TMBZ solution, 25 µL of 3% hydrogen peroxide, and 20 µL of the larval supernatant. Reactions were carried out in triplicate and incubated for 10 min at room temperature. Absorbance was measured at 620 nm using a microplate spectrophotometer. MFO enzymatic activity was expressed in nmol cytochrome equivalents/mg protein.

#### 2.6.5 α‐ and β‐Esterase Activity

A colorimetric method was used to evaluate α‐ and β‐esterase activities, based on the hydrolysis of specific naphthyl ester substrates, as described by Carreño Otero et al. [38]. In this assay, 10 µL of larval extract were added to 200 µL of phosphate buffer containing 0.3 mM α‐ or β‐naphthyl acetate. The enzymatic reaction proceeded for 15 min at ambient temperature. Afterward, 50 µL of a Fast Blue dye solution prepared by dissolving 30 mg of the dye in 3 mL of ultrapure water and mixing with 7 mL of a 3% SDS solution was added to each well to develop the color. Following a further 5‐min incubation, absorbance was read at 570 nm using a microplate reader. Each measurement was performed in triplicate, and enzyme activity was quantified as micromoles of α‐ or β‐naphthol produced per minute per milligram of total protein (µmol/min/mg protein).

#### 2.6.6 Acetylcholinesterase (AChE) Activity

AChE activity was evaluated using a modified version of the colorimetric method originally proposed by Ellman et al. [46], adapted for microplate format according to Abolaji et al. [42]. In each well of a 96‐well plate, a reaction mixture was assembled containing 135 µL of distilled water, 20 µL of 10 mM DTNB, 20 µL of phosphate buffer (100 mM, pH 7.3), 20 µL of an 8 mM solution of AChI, and 20 µL of the larval supernatant. The enzymatic hydrolysis of AChI produced a yellow chromophore, the formation of which was monitored by measuring absorbance at 412 nm every 30 s for a total duration of 2 min. All reactions were carried out in triplicate at 25°C, in the absence of light. AChE activity was calculated as the amount of substrate hydrolyzed per minute per milligram of protein and expressed in µmol/min/mg protein.

### 2.7 Assessment of Potential Lethal Effects of Bti Strain BR101 on Nontarget Insects

The potential lethal effects of the compound Bti strain BR101 on nontarget aquatic organisms were evaluated as described by Sivagnaname and Kalyanasundaram [47] and later adapted by de Oliveira et al. [7] under controlled temperature (28°C ± 2 °C) and relative humidity (80% ± 5%) conditions. Specimens from the families Notonectidae (*n* = 451) and Gerridae (*n* = 302), previously identified using a taxonomic key [48], were collected from the same breeding site as *Anopheles* spp. larvae using an entomological dipper.

The animals were separated by family and acclimated for 24 h in large containers (90 cm in diameter, 40 cm deep) filled with 5 L of water from their natural habitat. Subsequently, they were transferred to smaller containers (500 mL) containing 400 mL of habitat water and exposed to 74.80 µg/mL of Bti strain BR101. This concentration was obtained by multiplying the LC_90_ value (7.48 µg/mL) by a factor of 10. A DMSO control was prepared at the same concentration, and α‐cypermethrin was tested at 0.05 µg/mL (see Table 2).

The organisms were evenly distributed across the three treatments: approximately *n* = 150 Notonectidae and *n* = 100 Gerridae per treatment. The experiment was conducted over a 30‐day exposure period, during which survival curves were monitored to assess the potential lethal effects of the tested substances.

### 2.8 Statistical Analysis

Larvicidal activity percentages were analyzed using probit analysis in IBM SPSS Statistics to estimate LC_50_ and LC_90_ values, along with linear regression, chi‐square tests, and calculation of degrees of freedom. The estimated LC_50_ and LC_90_ values were further analyzed using the *t*‐test (*p* < 0.05). Enzymatic activity data were subjected to two‐way analysis of variance (ANOVA), with assumptions of normality and homogeneity of variance verified prior to analysis, followed by Tukey's post hoc test (*p* < 0.05) to identify statistically significant differences among treatment groups, using GraphPad Prism 9 software [49].

For survival data analysis, the Kaplan–Meier method was used to construct survival curves, and the log‐rank (Mantel–Cox) test was applied to compare them. Additionally, Cox regression analysis was performed to evaluate the influence of predictive factors (family and treatment). These analyses were also conducted using IBM SPSS Statistics [50].

## 3 Results

### 3.1 Larvicidal Assay

The larvicidal activity of Bti strain BR101 and α‐cypermethrin against *Anopheles* spp. larvae demonstrated clear dose‐dependent effects for both products. Linear regression analysis showed a strong relationship between the tested concentrations and observed mortality rates, with *R* ^2^ = 0.9257 (*F* = 37.35; *p* = 0.0088) for Bti and *R* ^2^ = 0.9709 (*F* = 133.3; *p* = 0.0003) for α‐cypermethrin.

**FIGURE 1:**
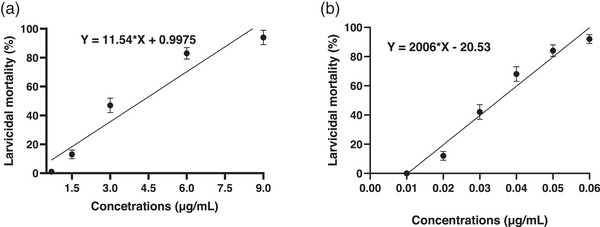
Mortality of *Anopheles* spp. larvae following 72 h exposure to Bti strain BR101 (a) and the positive control α‐cypermethrin (b). No mortality of larvae was observed in the negative control DMSO at concentrations ranging from 1.5 to 9 µg/mL.

In the bioassays, Bti strain BR101, evaluated at concentrations from 0.7 to 9 µg/mL, induced increasing mortality rates from 11%  ±  2% to 91%  ±  5%, with LC_50_ of 3.13 µg/mL and LC_90_ of 7.48 µg/mL. For α‐cypermethrin, at concentrations from 0.01 to 0.06 µg/mL, mortality ranged from 12% ± 3% to 92% ± 3% among 0.01 and 0.06 µg/mL, with LC_50_ of 0.03 µg/mL and LC_90_ of 0.05 µg/mL (Figure 1 and Table 1). Statistical comparison showed that both LC_50_ (*t* = 17.28; *p* < 0.0001) and LC_90_ (*t* = 11.11; *p* = 0.0004) values differed significantly among the two treatments. Therefore, on the basis of RP values, α‐cypermethrin was approximately 100 times more potent than Bti strain BR101 (RP = 1.0 vs. 0.010).

**TABLE 1:** Estimated lethal concentrations of Bti strain BR101 against *Anopheles* spp. larvae.

Sample	LC_50_ (µg/mL) (LCL–UCL)	LC_90_ (µg/mL) (LCL–UCL)	*χ* ^2^ (df)	*p* value^*^	Linear equation	Relative potency
Bti strain BR101	3.13^a^ (2.836–3.459)	7.48^a^ (6.522–8.869)	0.000 (3)^*^	0.151	*Y* = −1.684*x* + 3.394	0.010
α‐Cypermethrin	0.03^b^ (0.031–0.034)	0.05^b^ (0.052–0.062)	0.350 (4)^*^	0.2416	*Y* = −8.036*x* + 5.402	1

*Note*: LC_50_ and LC_90_—lethal concentrations to kill 50% and 90% of larvae. LCL—lower confidence limit of 95%. UCL—upper confidence limit of 95%. ^*^The *p* value obtained for *χ* ^2^ was not significant and corresponds to Pearson's goodness‐of‐fit test, with df representing the degrees of freedom. Different letters (a and b) within the same column indicate statistical differences, as determined by Student's *t*‐test (*t* = 17.28, df = 4, *p* < 0.0001 for LC_50_; *t* = 11.11, df = 4, *p* < 0.0001 for LC_90_).

### 3.2 Biochemical Assays

#### 3.2.1 Measurement of ROS

*Anopheles* spp. larvae exposed to Bti strain BR101 and α‐cypermethrin showed a marked increase in H_2_O_2_ levels compared to the DMSO control (ANOVA *F* (2,6) = 360.8; *p* < 0.0001; *R* ^2^ = 0.9918), indicating that both agents significantly disrupted redox homeostasis.

**FIGURE 2:**
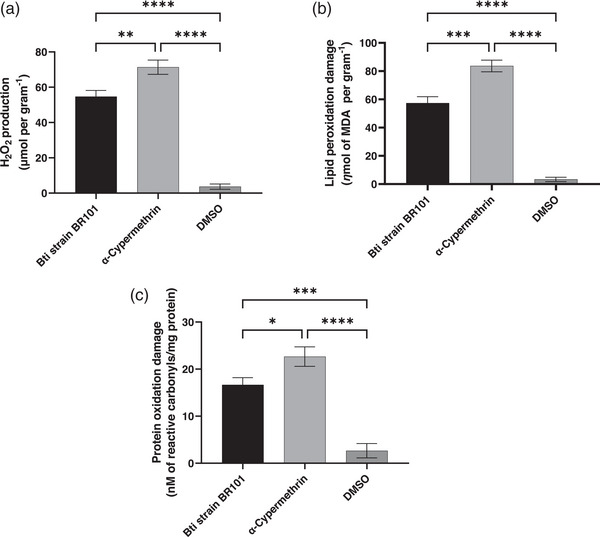
Production of reactive species, specifically H_2_O_2_ (a), along with the lipid peroxidation (b) and protein oxidation (c) damages in *Anopheles* spp. larvae exposure to Bti strain BR101 were compared with the control groups α‐cypermethrin and DMSO. Data are expressed as mean ± standard deviation. Data are presented as mean ± standard deviation. Statistical significance: (a) **p = 0.0010, ****p = 0.0001; (b) ***p = 0.0004, ****p = 0.0001; and (c) *p = 0.0400, ***p = 0.0007. DMSO, dimethyl sulfoxide; MDA, malondialdehyde.

Mean H_2_O_2_ concentrations reached 54.67 ± 3 µmol of H_2_O_2_ per gram for Bti strain BR101 and 71.33 ± 4 µmol of H_2_O_2_ per gram for α‐cypermethrin, whereas the control exhibited only 3.67 ± 1 µmol of H_2_O_2_ per gram. Moreover, α‐cypermethrin induced significantly higher ROS levels than Bti (*p* = 0.0017), suggesting a stronger oxidative challenge (Figure 2a).

#### 3.2.2 Lipid Peroxidation Damage

As shown in Figure 2b, lipid peroxidation levels, expressed as MDA concentration, closely followed the pattern observed for ROS production (*F* (2,6) = 377.4; *p* < 0.0001; *R* ^2^ = 0.9921), indicating a strong correlation between oxidative stress and membrane lipid damage. The highest MDA levels were recorded in larvae exposed to α‐cypermethrin (83.67 ± 4.0 ηmol/g), followed by those treated with Bti strain BR101 (57.33 ± 4.5 ηmol/g), both significantly higher than the control group (DMSO), which exhibited minimal lipid peroxidation (3.33 ± 1.5 ηmol/g; *p* = 0.0003).

These results highlight the pronounced oxidative impact of both larvicides, particularly the synthetic pyrethroid, on larval physiology. The elevated MDA levels in Bti‐treated larvae, although lower than those induced by α‐cypermethrin, suggest that even biological formulations can trigger measurable oxidative stress responses, potentially linked to the activation of immune or detoxification pathways.

#### 3.2.3 Protein Oxidation Damage

In addition to lipid peroxidation, oxidative damage to proteins was also significantly elevated in *Anopheles* spp. larvae, indicative of cellular stress responses, following exposure to the tested larvicides (*F* (2,6) = 105.3; *p* < 0.0001; *R* ^2^ = 0.9723). The formation of protein carbonyl groups was markedly higher in the group treated with α‐cypermethrin (22.67 ± 2 nM of reactive carbonyls/mg protein), followed by Bti strain BR101 (16.67 ± 1 nM of reactive carbonyls/mg protein), with a statistically significant difference between them (*p* = 0.0128).

In contrast, larvae exposed to DMSO showed only basal levels of protein oxidation (2.67 ± 1 nM of reactive carbonyls/mg protein), indicating negligible oxidative stress, as described in Figure 2c. The post hoc analysis confirmed significant pairwise differences across all treatments. These results reinforce that α‐cypermethrin exerts greater oxidative pressure on cellular macromolecules, extending beyond lipid peroxidation to include protein targets, whereas Bti, although less intense, also contributes to oxidative imbalance in exposed larvae.

### 3.3 Enzymatic Assay

#### 3.3.1 SOD Activity

The enzymatic activity of SOD, a key component of the antioxidant defense system, was significantly affected in *Anopheles* spp. larvae following exposure to Bti strain BR101 and α‐cypermethrin. The results revealed that larvae treated with α‐cypermethrin exhibited the highest SOD activity of 33.33 ± 3 mU/mg protein, followed by the Bti‐treated group of 29.00 ± 3 mU/mg protein, whereas the control group DMSO showed markedly lower enzyme activity of 10.33 ± 2 mU/mg protein (Figure 2a). Statistical analysis confirmed that these differences were highly significant (ANOVA, *F* (2,6) = 45.31; *p* = 0.0002; *R* ^2^ = 0.9379), indicating that the type of treatment substantially influenced SOD response.

Post hoc comparisons using Tukey's test showed that both Bti strain BR101 (*p* = 0.0008) and α‐cypermethrin (*p* = 0.0003) induced significantly higher SOD activity than DMSO, suggesting that both treatments triggered oxidative stress in the larvae. However, no significant difference was observed between the Bti strain BR101 and α‐cypermethrin groups (*p* = 0.2843), implying that although the chemical and biological agents differ in nature and mode of action, they both elicited a comparable antioxidant response.

#### 3.3.2 CAT Activity

**FIGURE 3:**
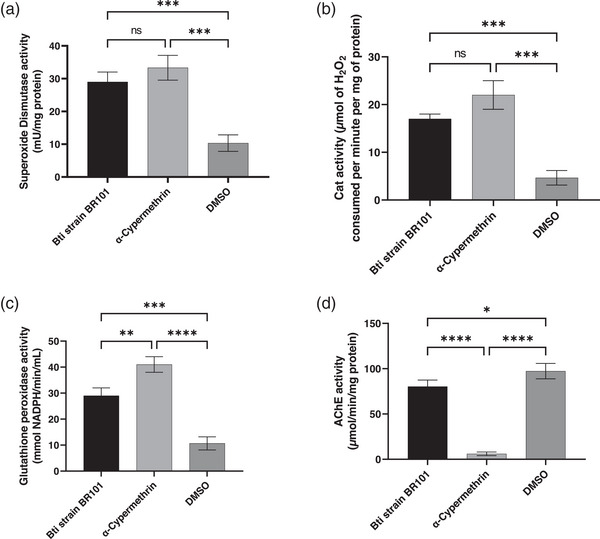
Changes in superoxide dismutase (a), catalase (b), and glutathione peroxidase (c), and acetylcholinesterase (AChE) activities in *Anopheles* spp. larvae exposure to Bti strain BR101 compared with the control groups α‐cypermethrin and DMSO. Data are expressed as mean ± standard deviation. Data are presented as mean ± standard deviation. Statistical significance: (a) **p = 0.0024, (b) ***p = 0.0014; (c) **p = 0.0170, ****p = 0.0001; (d) *p = 0.0471, ****p = 0.0001; ns = not significant. DMSO, dimethyl sulfoxide.

Exposure of *Anopheles* spp. larvae to the treatments resulted in significant changes in CAT activity, indicating an oxidative stress response. Indeed, larvae treated with α‐cypermethrin exhibited the highest enzymatic activity, reaching values of 22.00 ±  3 µmol of H_2_O_2_ decomposed per minute per mg of protein, followed by those exposed to Bti strain BR101, with values of 17.00 ± 1 µmol of H_2_O_2_ decomposed per minute per mg of protein. On the other hand, the control group (DMSO) showed markedly lower CAT activity, with 4.67 ± 1 µmol of H_2_O_2_ decomposed per minute per mg of protein. As described in Figure 3b.

Statistical analysis confirmed that these differences were significant (ANOVA, *F* (2,6) = 58.08; *p* = 0.0001; *R* ^2^ = 0.9509), indicating that the type of treatment had a substantial impact on this antioxidant system. Further, pairwise comparisons revealed that both α‐cypermethrin (*p* = 0.0001) and Bti strain BR101 (*p* = 0.0007) significantly increased CAT activity compared to DMSO. However, no statistically significant difference was observed between the Bti and α‐cypermethrin groups (*p* = 0.0531), suggesting that although the chemical insecticide induced a slightly stronger response, the overall magnitude of CAT activation was similar to that elicited by the biological agent.

#### 3.3.3 GPx Activity

Regarding GPx activity, the results in Figure 3d show that α‐cypermethrin also induced highest activity *Anopheles* spp. larvae, increasing in 41.00 ± 3 mmol NADPH/min/mL, indicating more toxicity than Bti strain BR101, that induced in 29.00 ± 3 mmol NADPH/min/mL, as well as the DMSO group, that showed the lowest activity of 10.67 ± 2 mmol NADPH/min/mL. Statistical analysis confirmed that the differences among the groups were highly significant (ANOVA, *F* (2,6) = 86.32; *p* < 0.0001; *R* ^2^ = 0.9664), indicating a strong effect of treatment type on the antioxidant response.

Post hoc analysis using Tukey's test revealed that both α‐cypermethrin (*p* < 0.0001) and Bti strain BR101 (*p* = 0.0005) induced significantly higher GPx activity compared to DMSO. Moreover, α‐cypermethrin also resulted in a significantly higher GPx level than Bti strain BR101 (*p* = 0.0050), suggesting that this chemical larvicide provoked a more intense oxidative stress, thereby increasing the enzymatic response in the exposed larvae.

#### 3.3.4 AChE Activity

Exposure of *Anopheles* spp. larvae to different treatments resulted in statistically significant variations in AChE activity, as indicated by analysis of variance (ANOVA), which revealed highly significant differences among groups (*F* (2,6) = 167.6; *p* < 0.0001; *R* ^2^ = 0.9824).

Larvae treated with α‐cypermethrin exhibited strong inhibition of AChE activity, with a mean value of 6.00 ± 2 µmol/min/mg protein, representing a drastic reduction compared to the control groups. Conversely, the group exposed to Bti strain BR101 showed intermediate AChE activity, reaching 80.33 ± 7 µmol/min/mg protein, which was significantly higher than that observed for α‐cypermethrin (*p* < 0.0001), but lower than the DMSO control group with value of 97.33 ± 8 µmol/min/mg protein, with a statistically significant difference between Bti strain BR101 and DMSO (*p* = 0.0423). Moreover, the comparison between α‐cypermethrin and DMSO revealed a marked difference (*p* < 0.0001), highlighting its pronounced impact on the cholinergic system of mosquito larvae.

The data analysis reinforces the distinction between the modes of action of the tested substances and underscores the disruptive potential of α‐cypermethrin on key neurotransmission enzymes such as AChE, which may be directly associated with its high larval toxicity observed in bioassays.

#### 3.3.5 MFO Activity

**FIGURE 4:**
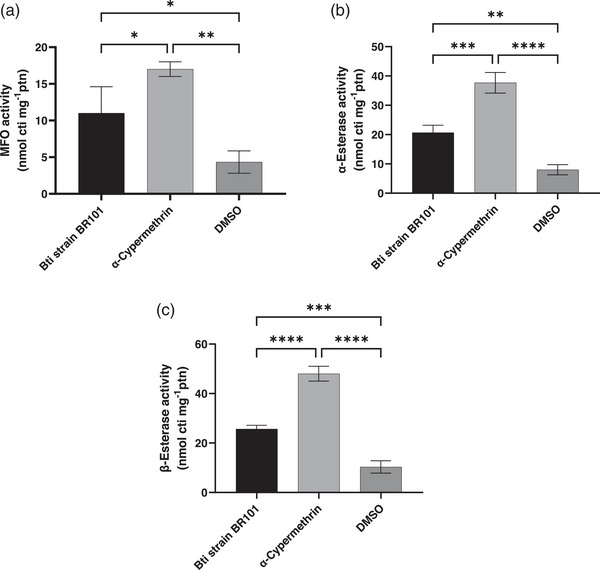
Alterations in mixed function oxidase (MFO) activity (a), α‐esterase activity (b), and β‐esterase (c) activity in *Anopheles* spp. larvae exposure to Bti strain BR101 compared with the control groups α‐cypermethrin and DMSO. Data are expressed as mean ± standard deviation. Data are presented as mean ± standard deviation. Statistical significance: (a) *p = 0.0123, **p = 0.0490; (b) **p = 0.0114, ***p = 0.0013; (c) ***p = 0.0070, ****p = 0.0001. DMSO, dimethyl sulfoxide.

The activity of MFO, enzymes involved in xenobiotic metabolism and detoxification, was significantly affected in *Anopheles* spp. larvae exposed to different treatments (Figure 4a). Larvae treated with α‐cypermethrin exhibited the highest enzymatic activity, with values reaching 17.00 ± 1 nmol cytochrome equivalents/mg protein, indicating a strong induction of the MFO system. The group treated with Bti also showed increased activity, with value of 11.00 ± 3 nmol cytochrome equivalents/mg protein, although to a lesser extent. Conversely, the control group exposed to DMSO presented the lowest MFO activity, with 4.33 ± 1 nmol cytochrome equivalents/mg protein, suggesting minimal stimulation of the detoxification pathway under normal conditions.

These observations were statistically supported by one‐way ANOVA, which revealed a significant effect of treatment on MFO activity (*F* (2,6) = 22.12; *p* = 0.0017; *R* ^2^ = 0.8806), indicating the variation in enzyme activity was explained by the treatment. Post hoc analysis using Tukey's multiple comparisons test confirmed that larvae exposed to α‐cypermethrin had significantly higher MFO activity than those treated with Bti strain BR101 (*p* = 0.0453) and DMSO (*p* = 0.0014). Additionally, Bti strain BR101 treatment also resulted in a moderate but statistically significant increase in activity compared to DMSO (*p* = 0.0297).

These findings suggest that both biological and chemical agents stimulate the oxidative metabolism in *An. darlingi* larvae, with α‐cypermethrin triggering a more robust activation of the MFO system. This response reflects the larvae's physiological attempt to metabolize and detoxify the xenobiotic compounds present in the treatments.

#### 3.3.6 α‐ and β‐Esterase Activity

The enzymatic activities of α‐ and β‐esterases in *Anopheles* spp. larvae varied significantly among the tested treatments, as demonstrated by one‐way ANOVA (α‐esterase: *F* (2,6) = 92.05, *p* < 0.0001, *R* ^2^ = 0.9684; β‐esterase: *F* (2,6) = 182.8, *p* < 0.0001, *R* ^2^ = 0.9839). These enzymes, known for their role in the detoxification of xenobiotics, responded differently depending on the compound to which the larvae were exposed.

For α‐esterase, larvae treated with α‐cypermethrin showed markedly elevated activity levels (37.67 ± 3 µmol/min/mg protein), significantly higher than both the Bti‐exposed group (20.67 ± 2 µmol/min/mg protein; *p* = 0.0006) and the DMSO control (8.00 ± 1 µmol/min/mg protein; *p* < 0.0001).

Similarly, β‐esterase activity was highest in the α‐cypermethrin group (48.00 ± 3 µmol/min/mg protein), followed by the Bti strain BR101 group (25.67 ± 1 µmol/min/mg protein; *p* < 0.0001), and lowest in the DMSO control (10.33 ± 2 µmol/min/mg protein; *p* < 0.0001). The differences between Bti strain BR101 and DMSO were also statistically significant for both enzymes (α‐esterase: *p* = 0.0029; β‐esterase: *p* = 0.0006).

These findings underscore the strong inductive effect of α‐cypermethrin and Bti strain BR101 on esterase activity; together, these results highlight the distinct biochemical responses elicited by synthetic and biological larvicides. Although α‐cypermethrin induces a strong esterase‐mediated detoxification response that could be linked to resistance development, Bti strain BR101 elicits only modest enzymatic changes, supporting its continued use as a low‐resistance‐risk alternative in integrated vector management.

### 3.4 Assessment of Potential Lethal Effects on Nontarget Insects

The survival analysis using the Kaplan–Meier model revealed significant differences among the treatments tested on aquatic predators of the orders Notonectidae and Gerridae. Individuals exposed to Bti strain BR101 and DMSO, both at a concentration of 74.80 µg/mL, exhibited 100% survival throughout the 30‐day experimental period. In contrast, the group treated with cypermethrin at 0.05 µg/mL exhibited 100% mortality across both orders, with a progressive decline until Day 30, reflecting the high toxicity. The log‐rank test indicated statistically significant differences among treatments (chi‐square = 20.654; df = 1; *p* < 0.001), confirming that cypermethrin severely compromised organism survival, whereas Bti strain BR101 and DMSO had no lethal effects.

**FIGURE 5:**
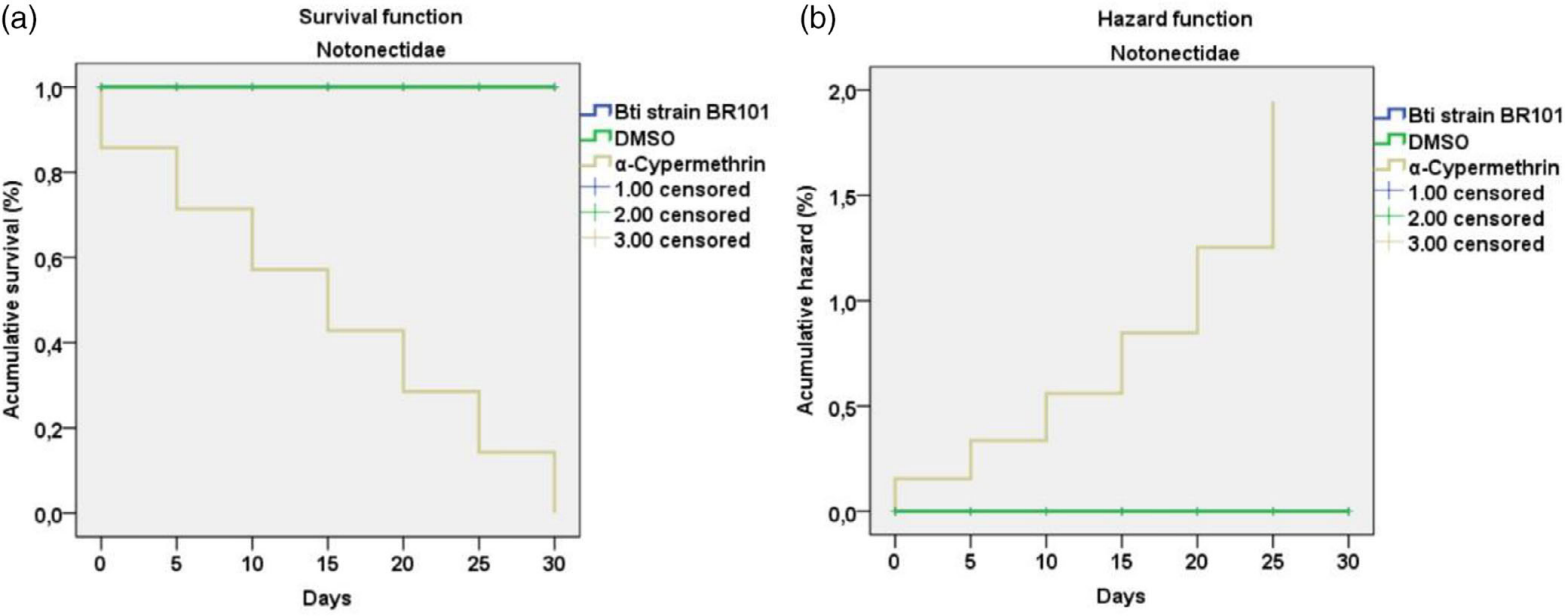
Survival (a) and cumulative hazard (b) curves for Notonectidae over a 30‐day period following exposure to Bti strain BR101, DMSO (74.80 µg/mL), and α‐cypermethrin (0.05 µg/mL). Analyses were performed using the Kaplan–Meier method, followed by the log‐rank test (Mantel‐Cox; chi‐square = 20.654; df = 1; *p* < 0.001). Censored data, meaning that no mortality was recorded at that point in the observation period. DMSO, dimethyl sulfoxide.

**FIGURE 6:**
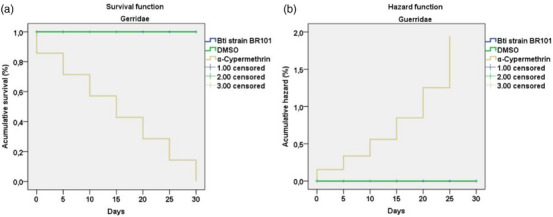
Survival (a) and cumulative hazard (b) curves for Gerridae over a 30‐day period following exposure to Bti strain BR101, DMSO (74.80 µg/mL), and α‐cypermethrin (0.05 µg/mL). Analyses were performed using the Kaplan–Meier method, followed by the log‐rank test (Mantel‐Cox; chi‐square = 20.654; df = 1; *p* < 0.001). Censored data, meaning that no mortality was recorded at that point in the observation period. DMSO, dimethyl sulfoxide.

The Cox regression analysis supported these findings, showing that the variable treatment significantly influenced mortality risk (chi‐square = 30.529; df = 1; *p* < 0.001). Exposure to cypermethrin resulted in a markedly increased risk of death (*B* = 11.360; *p* = 0.009; Exp(B) = 85 806.0) compared to Bti strain BR101 and DMSO. Conversely, the variables Notonectidae and Gerridae (families) showed no significant effect on mortality risk (*B* = 0.000; *p* = 1.000), indicating that both insect orders were equally susceptible to the lethal effect of cypermethrin and equally tolerant to the other treatments (Figures 5a,b and 6a,b) (Table 2). These results indicate that, at the tested concentrations, cypermethrin poses a lethal threat to the studied aquatic predators, whereas Bti and DMSO proved to be safe, not compromising the viability of these nontarget organisms.

**TABLE 2:** Cox proportional hazards model results for the effects of treatment and taxonomic order on the survival of aquatic predators over a 30‐day exposure period.

Variable	Coef. (B)	Standard error (SE)	Wald statistic	df	*p* value	Exp(B) (hazard ratio)	95.0% CI for Exp(B)	
							Low	Upper
Families	0.000	0.535	0.000	1.000	1.000	1.000	0.351	2.851
Treatment	11.360	78.287	0.021	0.009	0.021	85 806.037	0.000	3.729E + 071

*Note*: Order refers to the taxonomic group of the aquatic predators (Notonectidae vs. Gerridae). Treatment contrasts the effect of cypermethrin (0.05 µg/mL) with Bti and DMSO (both at 74.80 µg/mL), which did not result in any mortality. Coef. (B): regression coefficient. SE: standard error of the coefficient. Wald Statistic: used to assess the significance of individual predictors. df: degrees of freedom. *p* value: probability value for significance testing. Exp(B): hazard ratio, indicating the relative risk of death. 95% CI: 95% confidence interval for the hazard ratio.

## 4 Discussion

In Brazil, the Brazilian Ministry of Health recommends the use of the biological larvicides (as Bti‐based products) by targeting specific species of Diptera, because these products are considered an alternative for mosquito vector control, while minimizing harm to nontarget aquatic organisms. Thus, in a previous study, a new formulation based on Bti was developed and tested against *Cx. quinquefasciatus* larvae, showing excellent results for the control of this species [29].

This same formulation was adapted to verify its efficacy against *Anopheles* spp. The adaptation accounted for the surface‐feeding behavior of larvae, optimizing the release of Cry and Cyt toxins at the air–water interface where active ingestion occurs. Thus, the formulation was adjusted to ensure both stability and floatability, maintaining toxin availability within the larval feeding zone.

The larvicidal assays against *Anopheles* spp. revealed that Bti BR101 formulation achieved mortality rates exceeding 90% at concentrations below 10 µg/mL, with an LC_50_ of 3.13 µg/mL. These results confirm Bti strain BR101 is effective against *Anopheles* spp., aligning with the efficacy reported for other Bti strains against *Ae. aegypti*, *Ae. albopictus*, *Simulium* spp., and *Culex pipiens* (Culicidae) [51]. Moreover, its slower mode of action compared to neurotoxic insecticides may reduce selective pressure and delay the emergence of resistance in natural populations [28].

Bti acts through a complex mechanism involving Cry and Cyt proteins that bind to specific receptors in the midgut epithelial cells of mosquito larvae; upon ingestion, these toxins form pores in the gut epithelium, leading to cell lysis, disruption of the insect's midgut lining, and eventual death of the larvae [52]. This mode of action is highly selective, as nontarget organisms lack the necessary gut receptors, explaining the absence of mortality in predatory aquatic insects exposed to Bti strain BR101 in this study [53].

Importantly, the biochemical and enzymatic responses induced by Bti strain BR101 exposure provide further insights into its systemic effects. Indeed, although not directly, Bti strain BR101 triggered moderate oxidative stress in exposed *Anopheles* spp. larvae, as evidenced by increased hydrogen peroxide production and elevated lipid and protein oxidation [54]. These effects likely stem from cellular damage following midgut epithelial disruption, which secondarily leads to ROS generation and activation of stress response pathways [55]. However, the intensity of these responses was significantly lower than that induced by α‐cypermethrin, suggesting that Bti strain BR101 causes sublethal oxidative perturbations without overwhelming the larval redox balance [12].

Notably, the generation of ROS has been widely recognized as a mechanism of toxicity induced by entomopathogenic bacteria and insecticidal compounds [56]. Within the genus *Bacillus* (Bacillaceae), ROS induction has been described in detail for species such as *B. subtilis* Ehrenberg, 1835, *B. cereus* Frankland & Frankland, 1887, *B. thuringiensis* Berliner, 1915, and *B. anthracis* Cohn, 1872, which oxidative stress plays a key role in disrupting cellular homeostasis [57].

Similarly, ROS production has also been identified as a mechanism of action in studies involving natural insecticides derived from Piperaceae species. For instance, essential oils from *Piper alatipetiolatum* Yuncker [12], *Piper tuberculatum* Jacq. [17], and *Piper brachypetiolatum* Yuncker [18] have been shown to induce oxidative stress in *An. darlingi*, *Ae. aegypti*, *Cx. quinquefasciatus* Say, 1823 larvae, as have isolated phytocompounds such as 6‐ishwarona [58] and piplartine [16].

Additionally, pyrethroids are well known for their capacity to induce ROS, as reported against *Ae. aegypti* (103% ± 3%), *An. darlingi* (218% ± 1%) [16], and *Cx. quinquefasciatus* larvae (261% ± 3%) [12]. Indeed, in the present study, α‐cypermethrin induced significant ROS accumulation in larvae of *Anopheles* spp., supporting its known oxidative toxicity [11]. Similar mechanisms have been reported for other compounds such as imidacloprid [59], 4‐vinylcyclohexene 1,2‐monoepoxide, and 4‐vinylcyclohexene diepoxide [35], which trigger ROS‐related cellular damage in insects such as *Ae. aegypti* and *D. melanogaster* Meigen, 1830 (Drosophilidae).

Together, these findings reinforce the notion that ROS generation is a widespread cytotoxic mechanism shared across various classes of insecticidal agents, biological or chemical [60]. Regardless of origin, excessive ROS production leads to oxidative damage to key biomolecules such as lipids and proteins, impairing physiological function and ultimately resulting in larval death [61]. Recognizing this mechanism is essential for understanding the physiological basis of insecticidal activity and guiding the development of safer and more selective larvicides for vector control programs [55, 62].

This moderate oxidative stress induced by Bti strain BR101 was accompanied by the activation of the larvae endogenous antioxidant defense system, particularly involving enzymes such as SOD, CAT, and GPx. These enzymes act synergistically to neutralize ROS, maintaining redox balance and protecting cells from oxidative injury [28, 63].

SOD catalyzes the dismutation of superoxide radicals into hydrogen peroxide, which is subsequently decomposed by CAT and GPx into water and oxygen, thereby reducing cellular damage [58, 64]. The activation of these enzymes in Bti‐treated larvae indicates an adaptive response to moderate oxidative pressure, sufficient to trigger defense pathways without causing widespread cellular collapse [59, 62].

Interestingly, GPx activity was significantly higher in larvae treated with α‐cypermethrin, aligning with the elevated lipid peroxidation levels observed in that group [65]. GPx plays a central role in neutralizing lipid hydroperoxides, and its greater expression reflects a physiological response to the severe oxidative damage inflicted by the synthetic pyrethroid [66]. In contrast, the milder increase in GPx activity under Bti strain BR101 exposure further supports its selective toxicity and reduced physiological burden [65].

These differences reinforce Bti strain BR101 profile as a selective and biologically sparing larvicide [29]. Rather than inducing broad systemic damage, Bti strain BR101 elicits a targeted physiological disruption, focused on midgut epithelial integrity, with limited collateral oxidative stress [67]. This targeted mode of action preserves overall larval physiology until cell death is achieved, minimizing metabolic stress and promoting safety [53].

Moreover, Bti strain BR101 did not significantly inhibit AChE, a hallmark target of neurotoxic insecticides, whereas α‐cypermethrin exposure led to strong AChE inhibition, indicating direct neurotoxicity [68]. The preservation of AChE activity in Bti‐exposed larvae reinforces its non‐neurotoxic profile and its specificity for gut‐based mechanisms, distinguishing it mechanistically and ecologically from synthetic chemical insecticides [69].

The profile of detoxification enzyme activity further distinguishes Bti strain BR101 from α‐cypermethrin. Although the latter strongly induced MFOs and esterase biomarkers often associated with metabolic resistance, Bti strain BR101 elicited only modest changes in these enzymes [58]. This distinction is particularly relevant for resistance management, as overactivation of detoxification pathways is a key factor in the development of cross‐resistance to insecticides [70]. The limited biochemical disruption induced by Bti strain BR101 reduces selection pressure and favors its use in resistance mitigation strategies, including rotation with other larvicides and integration in multifaceted control programs [71].

Although Bti BR101 induced only modest changes in detoxification enzymes in laboratory assays, this suggests a lower likelihood of selecting for metabolic resistance in natural mosquito populations [34]. Nevertheless, continued monitoring in field populations is essential to confirm that repeated exposure does not promote gradual enzyme adaptation or reduced susceptibility over time [29].

In summary, the results of this study provide an integrated mechanistic understanding of the larvicidal activity of Bti BR101. Ingestion of the Bti BR101 formulation causes selective disruption of the midgut epithelium in *Anopheles* spp. larvae, triggering moderate production of ROS and oxidative modifications of lipids and proteins, which activate the endogenous antioxidant defenses without overwhelming the larval redox balance [68]. This controlled oxidative stress reflects a localized physiological disturbance that ultimately leads to larval death without inducing systemic damage or neurotoxicity [65].

In contrast, chemical insecticides such as α‐cypermethrin induce much higher ROS levels, cause intense lipid peroxidation, strongly activate detoxification enzymes, and inhibit AChE, resulting in widespread oxidative stress and systemic metabolic dysfunction [54], whereas α‐cypermethrin exerts broad, neurotoxic effects, and Bti BR101 acts specifically on the midgut, preserving overall larval physiology and minimizing metabolic and ecological impacts [48]. This mechanistic distinction underlies the selectivity, efficacy, and ecological safety of Bti BR101, as well as its potential for integration into resistance management strategies [59].

From an ecotoxicological perspective, the survival assays conducted with aquatic predators from the families Notonectidae and Gerridae provide compelling evidence that Bti strain BR101 is environmentally safety [67]. These hemipteran insects play critical roles in freshwater ecosystems as natural regulators of mosquito populations, contributing to the biological control of *Anopheles* spp. larvae through predation [26, 53]. In this study, no mortality was recorded in either taxon following exposure to Bti strain BR101, even at concentrations that were lethal to *Anopheles* spp. larvae, highlighting the high specificity and reinforcing its ecological compatibility [29].

In stark contrast, α‐cypermethrin at a concentration of 0.05 µg/mL caused 100% mortality in both nontarget taxa, emphasizing its broad‐spectrum toxicity and lack of ecological selectivity [72]. This finding is consistent with previous reports demonstrating the extreme sensitivity of various nontarget aquatic organisms to α‐cypermethrin, as documented in species such as *Toxorhynchites splendens* Wiedemann, 1819 (Culicidae), *Anisops bouvieri* Kirkaldy, 1904 (Notonectidae), *Gambusia affinis* Baird & Girard, 1853 (Poeciliidae), and *Diplonychus indicus* Venkatesan & Rao, 1980 (Belostomatidae), with LC_50_ values from 0.025 to 0.29 µg/mL [11, 16].

The Cox regression analysis clearly quantified this disparity in survival, revealing an extremely elevated hazard ratio for α‐cypermethrin exposure (Exp(B) = 85 806.0), whereas the insect order (predator identity) had no significant effect on susceptibility. These findings underscore the indiscriminate nature of synthetic, which, while effective against target organisms, can pose severe threats to beneficial fauna in aquatic food webs [73].

The use of larvicides that are toxic to nontarget species can lead to cascading ecological consequences, including the disruption of predator‐prey dynamics, reduction of natural mosquito control services, and imbalance of aquatic community structure [16, 74]. Predatory insects, like Notonectidae and Gerridae, are also important indicators of water quality and biodiversity; their elimination from breeding habitats may signal ecosystem degradation and facilitate mosquito resurgence in the absence of natural checks [73, 74].

In this context, Bti strain BR101 emerges as a model for environmentally responsible vector control. Its lack of toxicity to nontarget aquatic insects not only preserves the ecological integrity of breeding sites but also enhances the resilience and self‐regulation of aquatic ecosystems [75]. Maintaining predator populations is especially important in integrated mosquito management, where biological control is combined with larvicide application to ensure long‐term suppression of vector populations without compromising ecosystem health [76].

These results strongly support the prioritization of selective larvicides in vector control strategies, particularly in Brazil, where freshwater ecosystems harbor high biodiversity and where the excessive use of chemical insecticides has already contributed to significant environmental contamination and the emergence of insecticide resistance [77]. The adoption of microbial larvicides like Bti strain BR101 can mitigate these impacts, offering effective vector suppression while safeguarding the ecological services provided by aquatic invertebrates [78, 79].

## 5 Conclusion

This study provides the first comprehensive evidence of the mode of action and biological selectivity of the Bti strain BR101, specifically formulated to target *Anopheles* spp. larvae in the Amazon region. By integrating larvicidal efficacy, biochemical responses, and ecotoxicological safety, the findings not only deepen our understanding of this strain's selective mechanism but also establish as a promising and strategic tool for sustainable malaria vector control in the Amazon and beyond.

## Author Contributions

**Izabel Cristina de Oliveira Bentes**: conceptualization, data curation, formal analysis, investigation, methodology, original draft preparation, funding, and manuscript review and editing. **Dayane Dantas Abensour**: conceptualization, data curation, formal analysis, investigation, methodology, original draft preparation, funding, and manuscript review and editing. **Maria Luiza Lima da Costa**: conceptualization, data curation, formal analysis, investigation, methodology, original draft preparation, funding, and manuscript review and editing. **Raquel Telles de Moreira Sampaio**: methodological design, investigation, and formal data analysis. **Leticia Bernadete da Silva**: methodology development, formal analysis, data organization, drafting of the manuscript, and critical revision. **Francisco Augusto da Silva Ferreira**: methodological design, investigation, and formal data analysis. **Francisco de Assis Marque**: methodology development, formal analysis, data organization, drafting of the manuscript, and critical revision. **Mário Antonio Navarro da Silva**: methodology development, formal analysis, data organization, drafting of the manuscript, and critical revision. **Eduarda Andrade de Lima**: methodology development, formal analysis, data organization, drafting of the manuscript, and critical revision. **Gislayne Trindade Vilas‐Boas**: methodology development, formal analysis, data organization, drafting of the manuscript, and critical revision. **João Antonio Cyrino Zequi**: methodology development, formal analysis, data organization, drafting of the manuscript, and critical revision. **André Correa de Oliveira**: conceptualization, data curation, formal analysis, investigation, methodology, original draft preparation, funding, and manuscript review and editing. **Rosemary Aparecida Roque**: methodological design, investigation, and formal data analysis.

## Funding

The authors are grateful and declare having received a research grant from the Amazonas State Research Support Foundation (FAPEAM); Public Call Amazon +10 Initiative, Resolution No. 023/2022, 01.02.016301.04682/2022–87; Support and Innovation Program for Emerging Technologies—INOVATEC+, Call Resolution No. 015/2024. Furthermore, this study was supported by the Coordination for the Improvement of Higher Education Personnel (CAPES)—Finance code 001; State Secretariat for Economic Development, Science, Technology and Innovation (SEDECTI), and Government of Amazonas State.

## Conflicts of Interest

The authors declare no conflicts of interest.

## Data Availability

The data that support the findings of this study are available from the corresponding author upon reasonable request.
